# Current practice and perceptions of safety protocols for the use of intraperitoneal chemotherapy in the operating room: results of the IP-OR international survey

**DOI:** 10.1515/pp-2020-0148

**Published:** 2021-02-12

**Authors:** Daniel Clerc, Martin Hübner, K.R. Ashwin, S.P. Somashekhar, Beate Rau, Wim Ceelen, Wouter Willaert, Naoual Bakrin, Nathalie Laplace, Mohammed Al Hosni, Edgar Luis Garcia Lozcano, Sebastian Blaj, Pompiliu Piso, Andrea Di Giorgio, Giuseppe Vizzelli, Cécile Brigand, Jean-Baptiste Delhorme, Amandine Klipfel, Rami Archid, Giorgi Nadiradze, Marc A. Reymond, Olivia Sgarbura

**Affiliations:** Department of Visceral Surgery, Lausanne University Hospital (CHUV), University of Lausanne (UNIL), Lausanne, Switzerland; Department of Surgical Oncology and Robotic Surgery, Manipal Comprehensive Cancer Centre, Bengaluru, Karnataka, India; Department of Surgery, Campus Virchow-Klinikum and Charité Campus Mitte, Charité-Universitätsmedizin, Berlin, Germany; Department of Gastrointestinal Surgery, Ghent University Hospital Belgium, Gent, Belgium; Department of Digestive Surgery, Lyon-Sud University Hospital, Lyon, France; Department of Surgical Oncology, Cancer Institute of Montpellier (ICM), Montpellier, France; Department of General and Visceral Surgery, Krankenhaus Barmherzige Brüder, Regensburg, Germany; Peritoneum and Retroperitoneum Surgical Unit, Fondazione Policlinico Universitario A. Gemelli, IRCCS, Rome, Italy; Department of General and Digestive Surgery, Hautepierre Hospital, Strasbourg University Hospital, Strasbourg, France; Department of General and Transplant Surgery, University Hospital Tübingen and National Center for Pleura and Peritoneum, Tübingen, Germany

**Keywords:** environmental protection, personal protective equipment, PIPAC and HIPEC safety

## Abstract

**Objectives:**

To assess the risk perception and the uptake of measures preventing environment-related risks in the operating room (OR) during hyperthermic intraperitoneal chemotherapy (HIPEC) and pressurized intraperitoneal aerosol chemotherapy (PIPAC).

**Methods:**

A multicentric, international survey among OR teams in high-volume HIPEC and PIPAC centers: Surgeons (Surg), Scrub nurses (ScrubN), Anesthesiologists (Anest), Anesthesiology nurses (AnesthN), and OR Cleaning staff (CleanS). Scores extended from 0–10 (maximum).

**Results:**

Ten centers in six countries participated in the study (response rate 100%). Two hundred and eleven responses from 68 Surg (32%), 49 ScrubN (23%), 45 Anest (21%), 31 AnesthN (15%), and 18 CleanS (9%) were gathered. Individual uptake of protection measures was 51.4%, similar among professions and between HIPEC and PIPAC. Perceived levels of protection were 7.57 vs. 7.17 for PIPAC and HIPEC, respectively (p<0.05), with Anesth scoring the lowest (6.81). Perceived contamination risk was 4.19 for HIPEC vs. 3.5 for PIPAC (p<0.01). Information level was lower for CleanS and Anesth for HIPEC and PIPAC procedures compared to all other responders (6.48 vs. 4.86, and 6.48 vs. 5.67, p<0.01). Willingness to obtain more information was 86%, the highest among CleanS (94%).

**Conclusions:**

Experience with the current practice of safety protocols was similar during HIPEC and PIPAC. The individual uptake of protection measures was rather low. The safety perception was better for PIPAC, but the perceived level of protection remained relatively low. The willingness to obtain more information was high. Intensified, standardized training of all OR team members involved in HIPEC and PIPAC is meaningful.

## Introduction

Intraperitoneal (IP) chemotherapy administration has become part of the treatment options for peritoneal metastasis (PM). In the Western world, IP chemotherapy is applied, mainly intraoperatively. Hyperthermic Intraperitoneal Chemotherapy (HIPEC) commonly associated with cytoreductive surgery, is currently proposed for PM of different origins. More recently, the development of Pressurized Intraperitoneal Aerosol Chemotherapy (PIPAC) opened the path for the administration of IP chemotherapy as an aerosol for patients in palliative, neoadjuvant or adjuvant settings leading to rapid adoption of this procedure worldwide [1, 2].

Patient’s safety of IP administration during HIPEC and PIPAC has already been previously studied, with several reports demonstrating limited patient toxicity for both procedures [3], [4], [5], [6]. The occupational hazards of healthcare providers in this setting were mostly addressed in monocentric studies aiming to establish requirements for the manipulation of chemotherapy inside the operating room (OR). These reports confirmed the absence of air contamination, selective surface contamination, and selective contamination of the personal protective equipment [7], [8], [9], [10], [11], [12]. No evidence of occupational contamination based on blood or urine samples was reported [9, 12].

Despite concordant occupational hazard studies related to the intraoperative use of IP chemotherapy, applied OR safety protocols vary extensively [8], [13], [14], [15], [16]. Several professions are involved in the care of patients receiving IP chemotherapy in the OR, including surgeons, anesthesiologists, scrub nurses, and cleaning staff. Their perception concerning the safe use of chemotherapy in the OR as well as the requirements for training were not investigated so far. In this setting, OR safety during IP chemotherapy administration fits in the exploration [2b] phase of the IDEAL framework which can be evaluated through multi-institutional observational data.

We therefore aimed at addressing these issues through an international survey of all relevant professions of the OR team in high-volume HIPEC and PIPAC centers.

## Materials and methods

### Selection of the centers

An international survey was conducted among the OR team members of expert centers with a comprehensive practice in the surgical treatment of PM. Centers were considered eligible to participate in the study, provided they met the following criteria: (i) perform HIPEC and PIPAC procedures regularly, and (ii) have a cumulative experience of >50 HIPEC and >50 PIPAC procedures since the beginning of their PM surgical program.

### Study participants

An invitation was sent to the lead surgeon responsible for the PM program (Hospital lead) in each eligible center to distribute the survey to their co-workers: (i) Surgeons (Surg), (ii) Anesthesiologists (Anesth), (iii) Anesthesiology nurses (AnesthN), (iv) Scrub nurses (ScrubN), and (v) OR cleaning staff (CleanS), provided they were directly involved in the HIPEC and PIPAC procedures on a regularly basis. Completion of the survey by a minimum of two co-workers per category was encouraged. Hospital leads provided the local training standards to the investigators. All participating centers received authorship in the present study, for up to three collaborators per center, including the lead surgeon.

### Survey

The questionnaire included 18 closed-end questions covering four categories: (1) general information, (2) availability and current use of protection measures during HIPEC or PIPAC, (3) safety perception, and (4) prior information or education on the exposure risk. The questionnaire consisted of a revised version of the questionnaire previously used in a pilot single-center study [17]. Differences with the previous version included illustrations of the suggested safety measures to minimize subjectivity, and the questions were adapted to the practice of the whole OR team members (medical and non-medical). Moreover, a parallel design was conceived for HIPEC and PIPAC, to evaluate potential differences of practice between both procedures. The questionnaire was anonymous.

The investigators provided the centers with a printable form in English and French language. A set of 12 protective measures was proposed to the responders, who were asked to report if they systematically used each of these measures. Rate of systematic use of all measures was then calculated. When measures were not used systematically, the reasons for non-use were collected. Perceived availability of protective measures, degree of personal protection level, risk of contamination and Level of information were evaluated on a scale from 0 (min) to 10 (max). The full questionnaire is provided as supplementary material (Supplementary Material, Appendix 1).

### Statistical analysis

Data was analyzed centrally by the investigators, and center-specific results were shared with the hospital lead. No center-specific nor country-specific analyses were made but every center could use their data for internal quality control and feedback.

Chi-square and Student’s t-test were used for comparison of categorical and continuous variables, respectively. Statistical significance was accepted with p≤0.05.

## Results

The survey was conducted between March and July 2019. All eligible centers accepted participation in the study (response rate: 100%). Overall, there were 211 individual responders from 10 centers in six countries. Geographic distribution of the survey is detailed in Supplementary Material, Appendix 2. Median ‘responders’ count per center was 18 (range 12–54). Surg and ScrubN represented 55% of responders, with 68 (23%) and 49 (23%) respectively. Age of the responders was between 26 and 45 years for most of the responders (n=151, 72%). Experience of more than five years in the surgical treatment of PM was present in 45% (n=94). Complete demographics of the responders are shown in Table 1.

**Table 1:** Survey responder’s demographics.

	Overall	Surgeon	Anesthesist	Anesthesiology nurse	Surgical technician	Cleaning staff
Responders	211	68 (32%)	45 (21%)	31 (15%)	49 (23%)	18 (9%)
Gender female	114 (54%)	26 (38%)	17 (38%)	20 (65%)	39 (80%)	12 (67%)
**Age, years**						
18–25	10 (4.7%)	2 (3%)	0	0	6 (12%)	2 (11%)
26–35	91 (43%)	37 (54%)	15 (33%)	10 (32%)	23 (47%)	6 (33%)
36–45	60 (28%)	20 (29%)	13 (29%)	14 (45%)	10 (20%)	3 (17%)
46–55	37 (18%)	6 (9%)	14 (31%)	7 (23%)	8 (16%)	2 (11%)
>56	13 (6%)	3 (4%)	3 (7%)	0	2 (4%)	5 (28%)
**Experience, years**						
<1	28 (13%)	13 (19%)	3 (7%)	4 (13%)	6 (12%)	2 (11%)
<3	47 (22%)	22 (32%)	11 (24%)	1 (3%)	8 (16%)	5 (28%)
<5	42 (20%)	14 (21%)	7 (16%)	5 (16%)	13 (27%)	3 (17%)
>5	94 (45%)	19 (28%)	24 (53%)	21 (68%)	22 (45%)	8 (44%)

### Systematic use of protective measures

**Figure 1:**
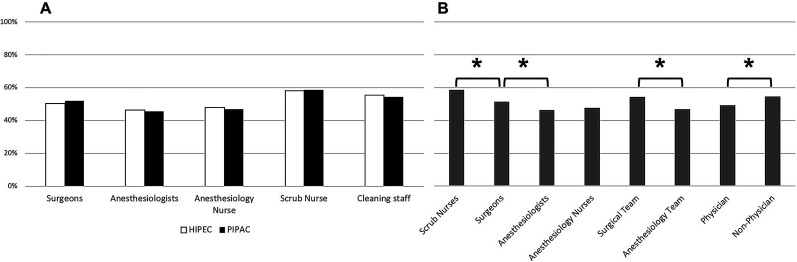
Comparison of systematic use of all protective measures, among type of procedure and professions. (A) Comparison between HIPEC and PIPAC, (B) Comparison among professions. Data outlined as percentages. *statistically significant difference. Surgical team: surgeons scrub nurses; anesthesiology team: anesthesiologists anesthesiology nurses; physician: surgeons anesthesiologists.

All protective measures were systematically used in 51.4% for HIPEC compared to 51.7% for PIPAC (p=0.88). The use of protective measures was similar among professions. Detailed data is outlined in Figure 1.

**Figure 2:**
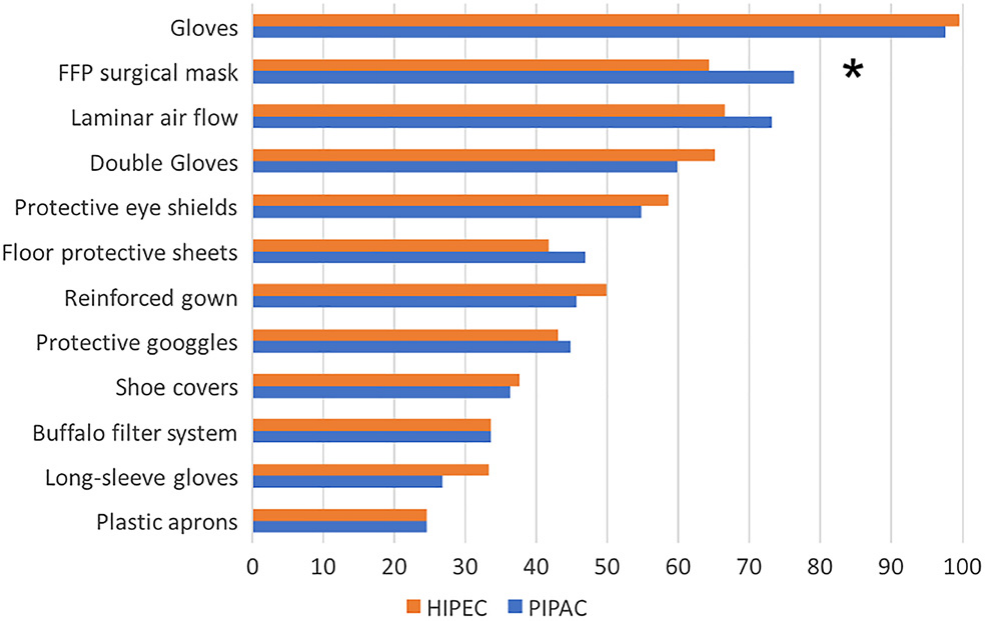
Detailed systematic use of protective measures. Data outlined as percentages. *Statistically significant difference.

There were no differences in the systematic use of individual protection measures between HIPEC and PIPAC, with one exception. The use of FFP 2/3 surgical masks was higher during PIPAC, compared to HIPEC (76 vs. 64% respectively, p<0.01). The overall detailed use of protective measures is shown in Figure 2. The main reasons for non-systematic use of potentially protective measures are detailed in Supplementary Material, Appendix 3.

### Safety perception

**Figure 3:**
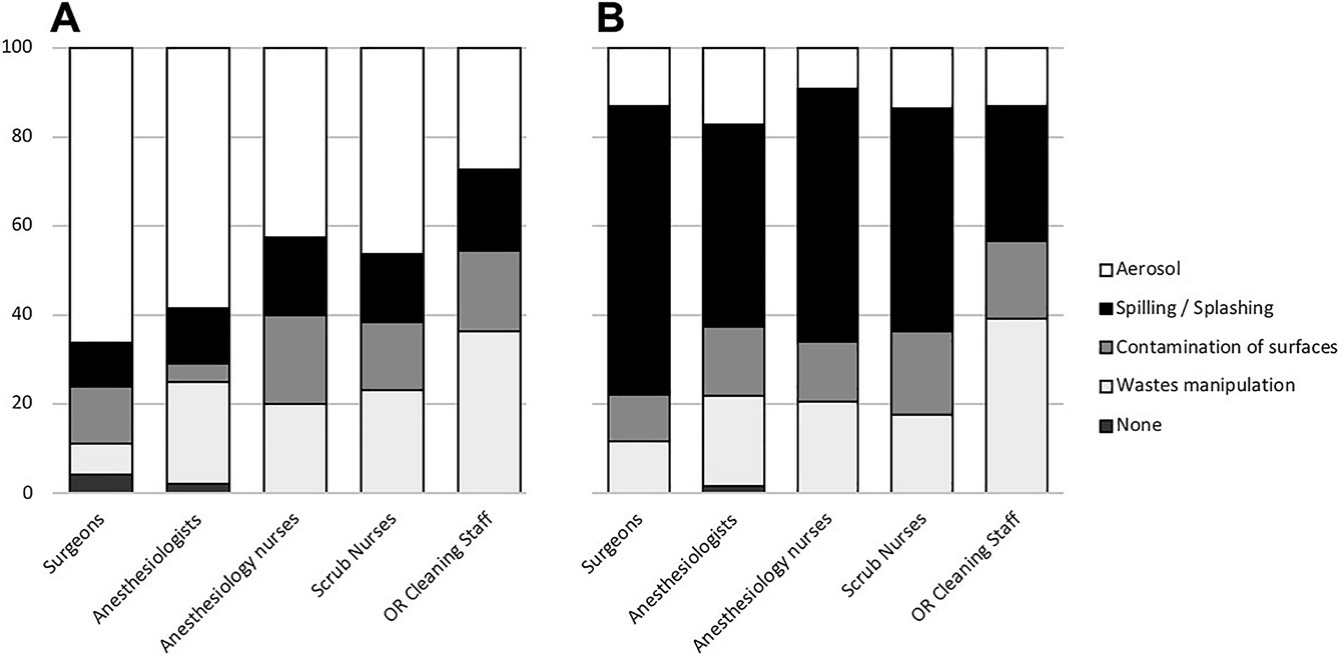
Perceived main risks of hazards. Data outlined as percentages. (A) PIPAC, (B) HIPEC.

The perceived main risks of intraoperative hazards due to exposure of chemotherapeutic agents were spilling/splashing of chemotherapy during HIPEC for 66% of the responders, and aerosol droplets during PIPAC for 63%. Detailed description of perceived main risks per profession, is shown in Figure 3.

**Figure 4:**
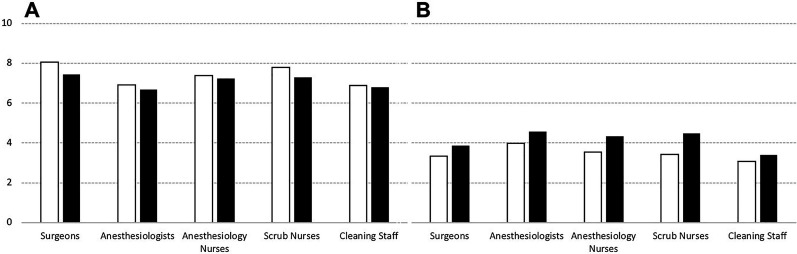
Perceived level of protection and contamination risk, per profession. Data outlined as 0–10 likert-scale, where 10 relates to greatest risk of contamination and highest protection level. PIPAC: white bars; HIPEC: black bars. (A) Level of protection, (B) Contamination risk.

Overall mean perceived degree of protection scored 7.37 (SD ± 0.195), higher during PIPAC, compared to HIPEC (7.57 ± 0.29 vs. 7.17 ± 0.26 respectively, p=0.045). The difference was more striking for Surg, who rated a higher degree of protection during PIPAC, compared to HIPEC (8.07 ± 0.40 vs. 7.44 ± 0.45 respectively, p=0.04), whereas the perceived degree of protection was similar among other professions. The Anesth mean degree of protection score was lowest among the professions, a difference statistically significant compared to the overall rating (6.81 ± 0.45 vs. 7.37 ± 0.195 respectively, p=0.02) (Figure 4).

The overall perceived risk of contamination level was 3.84 ± 0.224, higher for HIPEC compared to PIPAC (4.19 ± 0.30 vs. 3.5 ± 0.33 respectively, p<0.01). The ScrubN reported a higher risk of contamination during HIPEC, compared to PIPAC (4.47 ± 0.64 vs. 3.43 ± 0.65 respectively, p=0.03), but was otherwise similar between other professions (Figure 4).

Responders with over five years of experience in the surgical treatment of PM perceived lower risk of contamination and a higher level of protection, compared to the rest of the responders (contamination risk score: 3.1 vs. 4.1, p=0.03 and protection level score 7.6 vs. 7.2, p=0.02). Fifty-nine responders (28%) were aware of safety incidents during IP chemotherapy. They reported a median of one incident (range 1–10).

### Information and training

The overall mean level of information on the risk related to intraoperative manipulation of chemotherapy was 6.48 ± 0.243, similar between HIPEC and PIPAC (p=0.74) for all responders and among professions; Surg (p=0.61), Anesth (p=0.97), AnesthN (p=0.96), SurgT (p=0.93) and CleanS (p=0.86). However, Anesth and CleanS responders reported a lower level of information compared to all responders (5.67 ± 0.54, p<0.01 and 4.86 ± 0.89, p<0.01, respectively).

Participation in dedicated training on the required protective measures during HIPEC and PIPAC was reported for 42.6 and 50.5% of the responders, respectively, without statistically significant difference (p=0.11). The participation was similar among professions; Surg (p=0.06), Anesth (p=0.47), AnesthN (p=0.60), SurgT (p=1), CleanS (p=1). Surg reported participation in such training more frequently for PIPAC, compared to HIPEC (60.6 vs. 43.9% respectively), without reaching statistical significance (p=0.06). The overall rate was lowest for CleanS (22.2%) and Anesth (30.5%) and highest for ScrubN (56.3%) and AnesthN (55.2%). Among institutions, three centers offered only theoretical written support concerning safety, while five performed annual educational sessions with all caregivers for both procedures and two only for PIPAC. Willingness for supplementary information on the risk related to intraoperative chemotherapy administration was very high among all responders (85.6%), with 80.9, 84.1, 87.1, 89.8%, and respectively 94.1% for Surg, Anesth, AnesthN, ScrubN, and CleanS.

## Discussion

This international study investigated current practices and perceptions of safety protocols for IP intraoperative administration in 10 expert centers in PM. The main result is the rather low systematic uptake (51.4%) of all safety measures, taken to protect all persons involved. The practice was similar between HIPEC and PIPAC procedure and among different professions.

Our findings also outline the heterogeneity of safety protocols in place between centers. Only five out of 12 items were used systematically by >50% of responders. These items include gloves, double gloving, FFP 2/3 masks, eye shields, and advanced OR ventilation systems. Lack of standardization of safety protocols during HIPEC was already reported in a multicentric survey in France [16], while such data has not been published yet for PIPAC. Detailed use of protective measures was similar between HIPEC and PIPAC, except FFP-masks, which were used more frequently during PIPAC. This use is explained mainly by the perceived need the prevent accidental inhalation of aerosolized chemotherapy. However, very low to inexistent air contamination after PIPAC has been measured [10, 18]. Other potentially protective measures were not used because of unavailability (30%) or because these measures were not included in the respective institutional protocol (29%). In our survey, oversight and discomfort were rarely reported, unlike previous report [17].

The surgical team had higher use of protective measures compared with the anesthesiology team. This is not surprising since the perceived risk is higher for persons in direct contact with chemotherapy. Nevertheless, non-physicians adhered more largely to safety measures compared to physicians. For example, the use of protective measures by the ScrubN was higher than by Surg. Awareness of occupational hazards with a high level of trust in the efficacy of the safety measures during the IP administration of non-medical caregivers [17] could explain this finding. Previous reports also suggested a lower level of compliance of physicians to safety measures, as documented by hand hygiene audits [19].

Considering IP administration as a potential occupational risk, we found somehow low overall safety perception levels (protection level 7.37, contamination risk 3.84, level of information 6.48). PIPAC safety perception scored statistically significantly better regarding protection level and contamination risk, but with only slight absolute differences in the reported score (*protection level:* 7.57 vs. 7.17 and *contamination risk*: 4.19 vs. 3.5). The latter could be explained by the fact that PIPAC is a newer, minimally invasive, highly standardized minimally invasive procedure, with more recent training of the OR team members and mandatory training courses with an emphasis on safety checklists [20].

Anesth reported a lower perceived level of protection, information, and lower rates of participation in dedicated training. Anesth might not have been included in specialized training for HIPEC or PIPAC, leading to less information, a higher perception of risk and, thus, a lower perceived level of protection. Another explanation could be that specific dedication to surgical treatment of PM is probably more frequent among Surg leading to a higher turnover rate in large teaching hospitals among Anesth caring for PM patients.

Training among institutions was heterogeneous. Only half of the centers surveyed included educational sessions for both HIPEC and PIPAC procedures, with differences in training medical and non-medical personnel. PIPAC training for Surg is largely standardized through the collective work of the members of the International Society for the Study of Pleura and Peritoneum (ISSPP) [21]. The standardized PIPAC training might explain the higher safety perception scores reported in this survey and highlights the need to extend standardized periodic training to all persons involved in the care of patients undergoing intraoperative IP administration.

CleanS reported the lowest level of information and participation in dedicated training, and the highest reported willingness to obtain supplementary information. In contrast, protection level and contamination risk scores were not higher. These results outline the central role of communication within the entire OR team and the need for structured information and training for all members.

Some limitations of the present study must be addressed. The survey results are limited by the lack of control of the current practice protocols in place within centers, as the survey evaluated individual self-declaration of dedicated PM surgery co-workers, and there was no independent audit of local practice. We aimed at inviting all PM centers worldwide with significative experience in both HIPEC and PIPAC, but omissions cannot be excluded. A potential change in safety practices among participating centers could not be captured in this snapshot survey. After establishment of standardized consensus safety recommendations, a follow-up survey could address this issue.

In conclusion, the current safety practice during IP administration in expert PM centers appears similar between HIPEC and PIPAC, but overall systematic use of protective measures seems not standardized enough. Consensus guidelines on safety practices during HIPEC and PIPAC should be established, along with regular dedicated safety training, including all different professions of the OR team. Continuous information and communication on the risks and safety protocols related to IP administration of chemotherapy is advised to enhanced co-worker’s safety perception.
