# Supplementary material for: Current practice and perceptions of safety protocols for the use of intraperitoneal chemotherapy in the operating room: results of the IP-OR international survey
Source: Pleura Peritoneum. 2021 Feb 12;6(1):39–45. doi: 10.1515/pp-2020-0148 (PMC8223803; doi:10.1515/pp-2020-0148)
Supplement: Supplementary file 1 [file pp-06-20200148-s001.pdf]

Survey on current practice and perceptions of safety protocols for the use of intraperitoneal chemotherapy

This is a survey on safety measures and perceived safety among health professionals involved in the treatment of peritoneal cancer patients by HIPEC (*Hyperthermic intraperitoneal chemotherapy)* and PIPAC (*Pressurized intraperitoneal aerosol chemotherapy*). Aim of this study is not only to assess current practice but also to analyze feelings of safety/danger in all professional groups involved in order to confirm and optimize the present protocols.

Your contribution is very much appreciated, thank you for your time!

General Information

1) Gender

□ Female □ Male

2) Age

□ 18-25 □ 26-35 □ 36-45 □ 46-55 □ >56

3) Role

□ Surgeon

□ Anesthesiologist

□ Anesthesiology nurse

□ Surgical technician / Scrub nurse / Operating room circulating nurse

□ Operating room cleaning staff

4) Professional experience on the manipulation, elimination and caution of chemotherapeutic agents (by direct or indirect contact)

□ < 1 year □ < 3 years □ < 5 years □ > 5 years

Availability of protective measures/devices

5) In your institution, to which extent are there protective measures/devices available for peritoneal cancer surgery (HIPEC / PIPAC)? *Rate between 0 to 10. (0=insuffisant; 10= optimal)*

1 2 3 4 5 6 7 8 9 10

6) Which of the protective measures below do you systematically use in your institution during peritoneal cancer surgery? *Please encircle your answer for HIPEC and PIPAC for each item listed.*

Device	HIPEC			PIPAC		
Gloves	Yes	No	I don't know	Yes	No	I don't know
Double Gloves	Yes	No	I don't know	Yes	No	I don't know
Long-sleeve gloves	Yes	No	I don't know	Yes	No	I don't know
Protective eye shields	Yes	No	I don't know	Yes	No	I don't know
Protective goggles	Yes	No	I don't know	Yes	No	I don't know
Shoe covers	Yes	No	I don't know	Yes	No	I don't know
FFP surgical mask	Yes	No	I don't know	Yes	No	I don't know
Reinforced gown	Yes	No	I don't know	Yes	No	I don't know
Plastic aprons	Yes	No	I don't know	Yes	No	I don't know
Floor protective sheets	Yes	No	I don't know	Yes	No	I don't know
Laminar air flow	Yes	No	I don't know	Yes	No	I don't know
Buffalo filter system	Yes	No	I don't know	Yes	No	I don't know

7) Can you explain why you don't use some or all of the above-mentioned devices? *Please encircle your answer for both procedures whether HIPEC or PIPAC for each device listed*.

a) During HIPEC

Device	HIPEC					
Gloves	Not available	Not useful	I forget	I'm not at risk	Uncomfortable	Not in protocol
Double Gloves	Not available	Not useful	I forget	I'm not at risk	Uncomfortable	Not in protocol
Long-sleeve gloves	Not available	Not useful	I forget	I'm not at risk	Uncomfortable	Not in protocol
Protective eye shields	Not available	Not useful	I forget	I'm not at risk	Uncomfortable	Not in protocol
Protective goggles	Not available	Not useful	I forget	I'm not at risk	Uncomfortable	Not in protocol
Shoe covers	Not available	Not useful	I forget	I'm not at risk	Uncomfortable	Not in protocol
FFP surgical mask	Not available	Not useful	I forget	I'm not at risk	Uncomfortable	Not in protocol
Reinforced gown	Not available	Not useful	I forget	I'm not at risk	Uncomfortable	Not in protocol
Plastic aprons	Not available	Not useful	I forget	I'm not at risk	Uncomfortable	Not in protocol
Floor protective sheets	Not available	Not useful	I forget	I'm not at risk	Uncomfortable	Not in protocol
Laminar air flow	Not available	Not useful	I forget	I'm not at risk	Uncomfortable	Not in protocol
Buffalo filter system	Not available	Not useful	I forget	I'm not at risk	Uncomfortable	Not in protocol

b) During PIPAC

Device	PIPAC					
Gloves	Not available	Not useful	I forget	I'm not at risk	Uncomfortable	Not in protocol
Double Gloves	Not available	Not useful	I forget	I'm not at risk	Uncomfortable	Not in protocol
Long-sleeve gloves	Not available	Not useful	I forget	I'm not at risk	Uncomfortable	Not in protocol
Protective eye shields	Not available	Not useful	I forget	I'm not at risk	Uncomfortable	Not in protocol
Protective goggles	Not available	Not useful	I forget	I'm not at risk	Uncomfortable	Not in protocol
Shoe covers	Not available	Not useful	I forget	I'm not at risk	Uncomfortable	Not in protocol
FFP surgical mask	Not available	Not useful	I forget	I'm not at risk	Uncomfortable	Not in protocol
Reinforced gown	Not available	Not useful	I forget	I'm not at risk	Uncomfortable	Not in protocol
Plastic aprons	Not available	Not useful	I forget	I'm not at risk	Uncomfortable	Not in protocol
Floor protective sheets	Not available	Not useful	I forget	I'm not at risk	Uncomfortable	Not in protocol
Laminar air flow	Not available	Not useful	I forget	I'm not at risk	Uncomfortable	Not in protocol
Buffalo filter system	Not available	Not useful	I forget	I'm not at risk	Uncomfortable	Not in protocol

8) Do you have access to a safety monitoring system in your hospital *(CIRS – Critical Incident Reporting System)*?

□ yes □ no □ I don't know

9) Are you aware of any incidents which occurred during HIPEC or PIPAC?

□ yes □ no

a) How many occurred? ___________________________

b) How many were reported? ___________________________

10) In case of exposure of a surgical team member by chemotherapeutic agents during peritoneal cancer surgery, do you have access to a dedicated emergency spillage kit in your institution?

□ yes □ no □ I don't know

Safety perception

11) What do you think being the main risk of hazard due to chemotherapeutic agents during HIPEC and PIPAC? *Please cross the single most appropriate answer in each column.*

Main risk of hazard	HIPEC	PIPAC
Aerosols droplets		
Spilling or splashing of chemotherapy		
Contamination of surfaces		
Manipulation of contaminated wastes		
None		

12) During HIPEC and PIPAC procedures, how do you rate the protection measures/devices available in your institution? *Please cross the single most appropriate answer in each column.*

Amount of protective measure/devices required	HIPEC	PIPAC
Adequate		
Exaggerated		
Incomplete		
Inexistant		

If you didn't answer *"Adequate"* above, please comment why:

For HIPEC:
………………………………………………………………………………………………………
………………………………………………………………………………………………………

For PIPAC:
……………………………………………………………………………………………………..
……………………………………………………………………………………………………..

13) How do you generally rate your risk of contamination due to chemotherapeutic agents during HIPEC and PIPAC? *Rate between 0 to 10. (0=no risk; 10= high risk)*

For HIPEC:

1 2 3 4 5 6 7 8 9 10

For PIPAC:

1 2 3 4 5 6 7 8 9 10

14) How strong do you rate your personal protection level in regards to the manipulation (by direct or indirect contact) of chemotherapeutic agents during or after surgery. *Rate between 0 to 10. (0=not at all protected; 10= completely protected)*

During HIPEC:

1 2 3 4 5 6 7 8 9 10

During PIPAC:

1 2 3 4 5 6 7 8 9 10

Information / education

15) Have you received dedicated training on the protective measures/devices required during HIPEC?

□ yes □ no

16) Have you received dedicated training on the protective measures/devices required during PIPAC?

□ yes □ no

17)How do you rate your level of information on the risks of chemotherapy related hazards of the operating room staff during HIPEC procedures? Rate between 0 to 10. (0= no information; 10=fully informed)

For HIPEC:

1 2 3 4 5 6 7 8 9 10

For PIPAC:

1 2 3 4 5 6 7 8 9 10

Would you like to receive more information on the risks of chemotherapy related hazards of the operating room staff during HIPEC or PIPAC procedures?

□ yes □ no

Comments / Suggestions:

…………………………………………………………………………………………………
…………………………………………………………………………………………………
…………………………………………………………………………………………………
…………………………………………………………………………………………………
…………………………………………………………………………………………………..
…………………………………………………………………………………………………

Thank you for participation!

Appendix 1: Illustration of the material / devices mentioned on Q6 + Q7a-b

a) **Gloves**

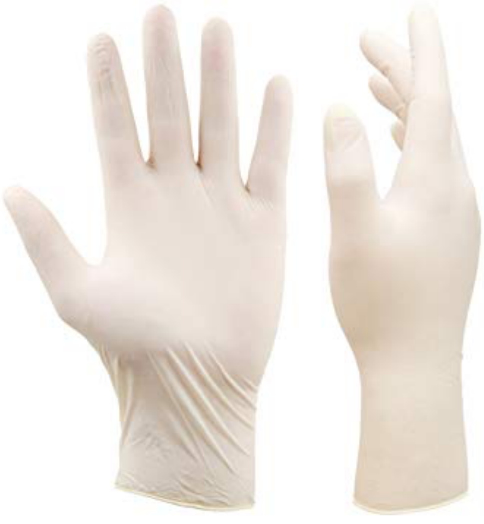

b) **Double Gloves refers to wearing two pairs of a)**

c) **Long-sleeve gloves**

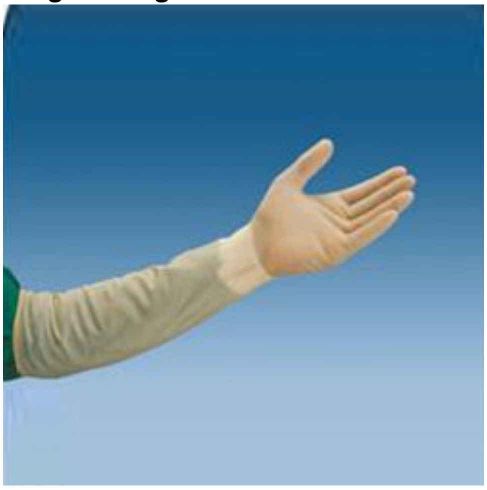

d) Protective eye shields

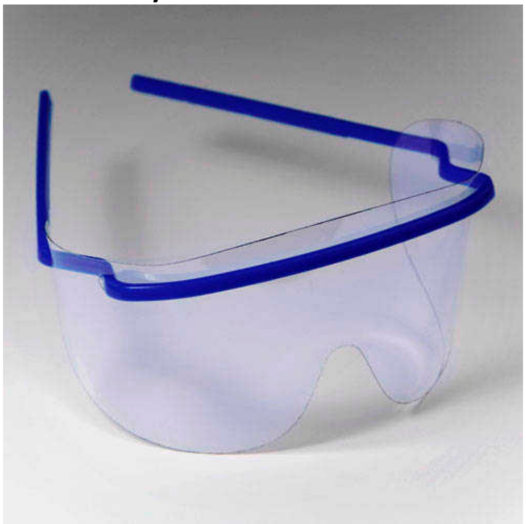

e) Protective goggles

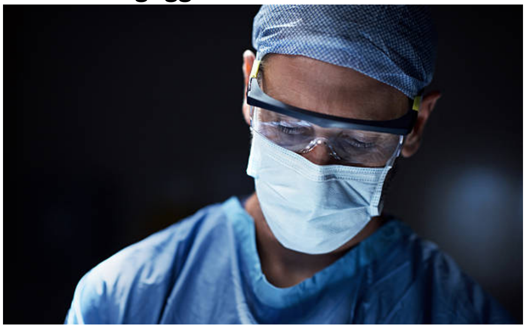

f) Shoe covers

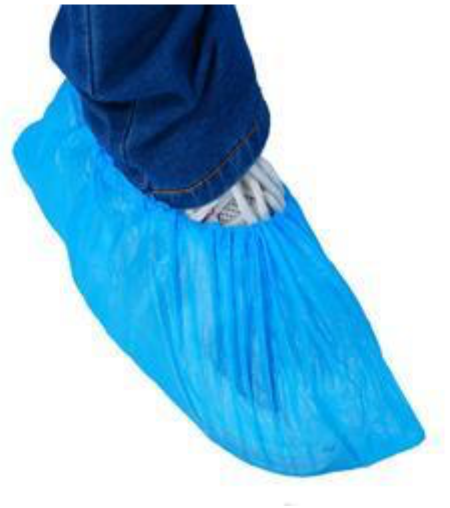

g) FFP surgical mask

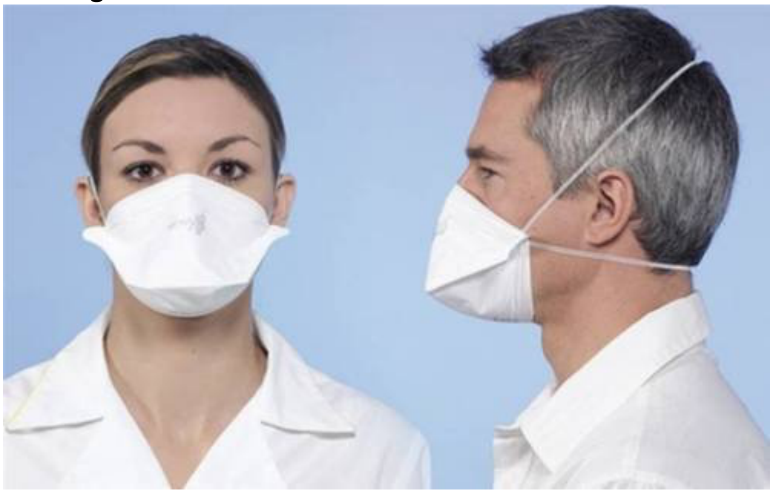

FFP2

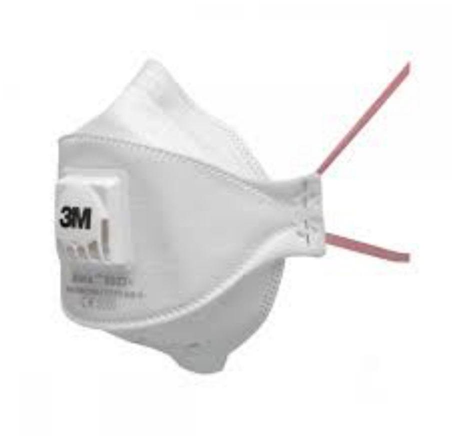

FFP3

h) Reinforced gown

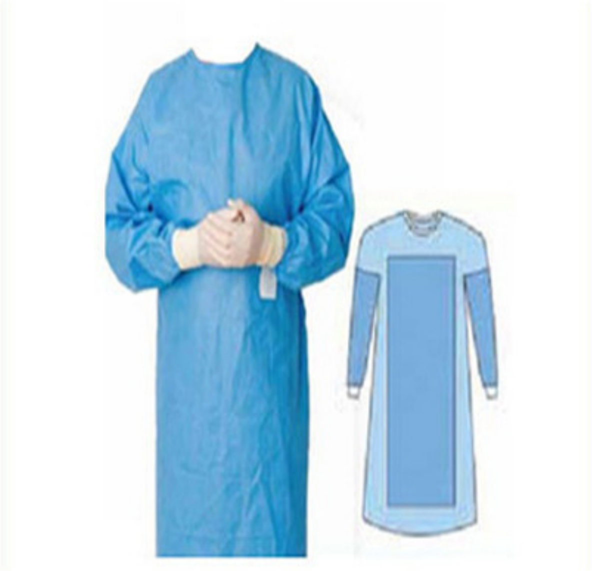

i) Plastic aprons

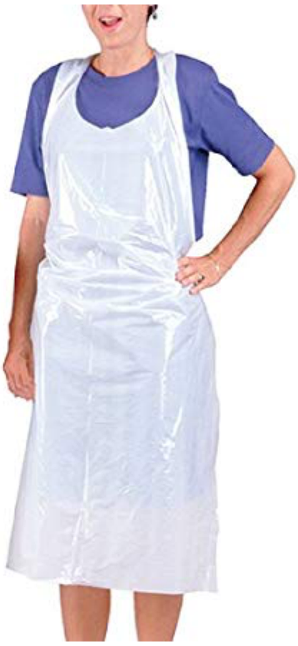

j) Floor protective sheets

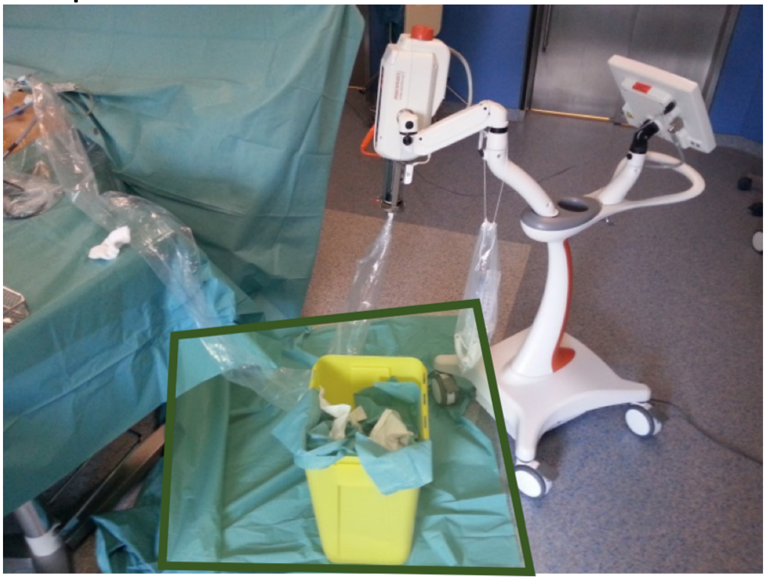

k) Laminar air flow

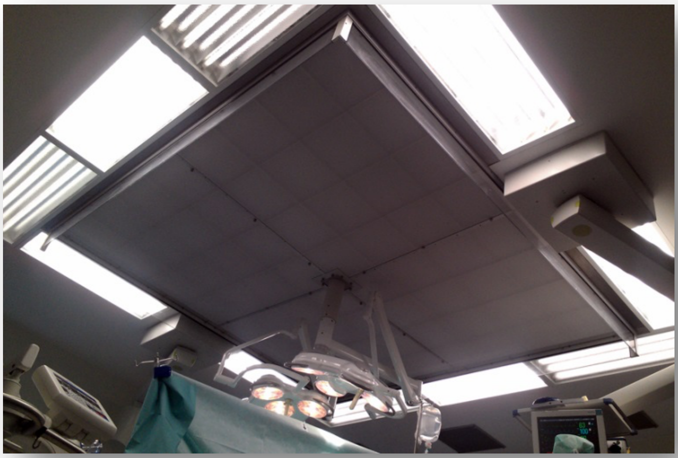

l) Buffalo filter system or other surgical gas aspiration systems (ERBE, Covidien etc)

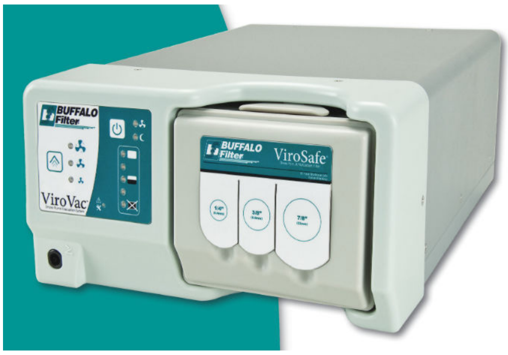

Buffalo

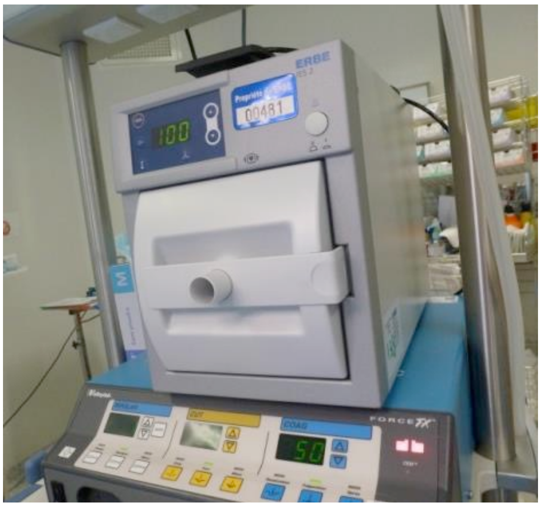

ERBE
