# Supplementary material for: Current practice and perceptions of safety protocols for the use of intraperitoneal chemotherapy in the operating room: results of the IP-OR international survey
Source: Pleura Peritoneum. 2021 Feb 12;6(1):39–45. doi: 10.1515/pp-2020-0148 (PMC8223803; doi:10.1515/pp-2020-0148)
Supplement: Supplementary file 2 [file pp-06-20200148-s002.docx]

**Supplementary Appendix 2:** Geographic survey distribution

**
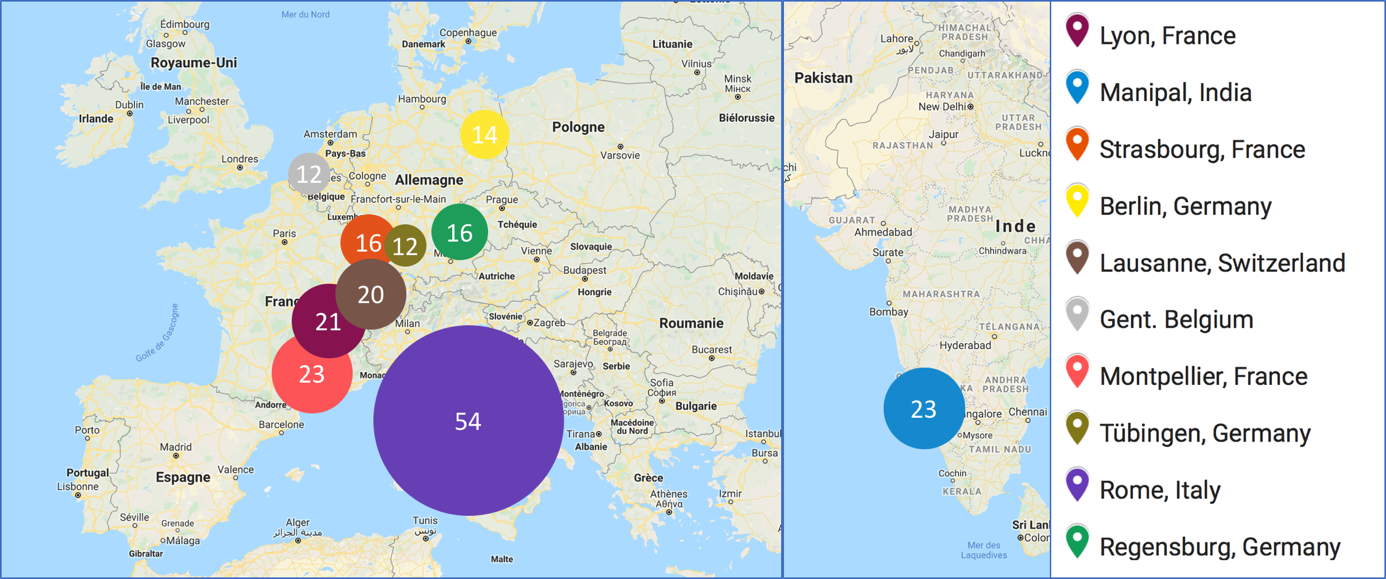
**

Data is outlined as number of survey participant, along with sizing cricles

**Supplementary Appendix 3 :** Reasons not to use potentially protection measures.

|  | HIPEC | PIPAC | p-value |
| --- | --- | --- | --- |
| Not available | 31.4% | 27.6% | **0.04** |
| Not in protocol | 29.7% | 29.2% | 0.79 |
| Not useful | 11.4% | 11.2% | 0.84 |
| Not at risk | 10.4% | 11.1% | 0.53 |
| Uncomfortable | 2.9% | 2.6% | 0.63 |
| Oversight | 3.5% | 2% | **0.03** |
| No Explantation | 10.8% | 16.6% | **<0.01** |

*Data outlined in percentages of overall mean response.*
